# The effect of ketorolac on biofilm of *Staphylococcus epidermidis* isolated from post-cataract endophthalmitis

**DOI:** 10.1007/s12348-012-0070-1

**Published:** 2012-03-13

**Authors:** Silvia Rossetti, Leonardo D’Alessandro, Fernando Pellegrino, María Alejandra Carrasco

**Affiliations:** 1 Infectious Diseases Unit, Fundación Oftalmológica Argentina Jorge Malbrán, Parera 164, C1014ABD Ciudad Autónoma de Buenos Aires, Argentina; 2 Department of Ophthalmology, Hospital de Clínicas “José de San Martín”, Universidad de Buenos Aires, Av. Córdoba 2351, C1120AAR Ciudad Autónoma de Buenos Aires, Argentina; 3 Department of Ophthalmology, Hospital Universitario de Mendoza, Paso de los Andes 3051, M5502BLI Mendoza, Argentina; 4 Sarmiento 1326. Luzuriaga, M5516BGH Mendoza, Argentina

**Keywords:** Ketorolac, NSAIDs, Endophthalmitis, Biofilm, *Staphylococcus epidermidis*

## Abstract

**Purpose:**

The purpose of this study is to investigate the effect of ketorolac on biofilm formation of *Staphylococcus epidermidis* isolated from patients with post-cataract endophthalmitis.

**Methods:**

Forty *S. epidermidis* strains isolated from postoperative endophthalmitis were used for this study. Biofilms were grown on microtitre plates for 24 h, dyed, and stained with crystal violet. The mean optical density (OD) and the OD ratio (ODr = OD of the treated biofilm/OD of the untreated biofilm) were used for quantification. The biofilms were incubated with 13 mM ketorolac and without ketorolac for controls.

**Results:**

The biofilm ODs of the *S. epidermidis* isolates untreated and treated with ketorolac were significantly different (0.335 ± 0.06 versus 0.158 ± 0.03, respectively; mean ± SD; *P* < 0.001). Ketorolac reduced *S. epidermidis* biofilm formation by 47.6 %.

**Conclusions:**

Ketorolac, at a concentration of 13 mM, significantly reduces the formation of biofilm by strains of *S. epidermidis* that caused endophthalmitis.

## Introduction

Endophthalmitis is a devastating sight-threatening postsurgical ocular complication. It remains the major concern for all cataract surgeons. Trends in favorable pre- and perioperative prophylaxis practices are difficult to analyze because of the infrequent occurrence of postoperative infection.

*Staphylococcus epidermidis* is recognized as the most common organism isolated in post-cataract endophthalmitis [1]. We previously found that 75 % of our *S. epidermidis* post-cataract endophthalmitis strains had the genotypic markers of virulence (*ica*A, *ica*D, and *mec*A) and were biofilm producers [2].

Nonsteroidal anti-inflammatory drugs (NSAIDs) like sodium salicylate decrease the production of biofilm and bacterial adhesion to contact lenses [3, 4]. Considering that ketorolac is commonly used to prevent ocular inflammation, pain, and miosis in the perioperative period of cataract surgery, we designed an in vitro study to investigate the effect of ketorolac on the formation of biofilm by *S. epidermidis*.

## Methods

### Microorganisms

The present study included 40 *S. epidermidis* strains isolated from postoperative endophthalmitis between the years 2000 and 2008. All isolates were initially identified by classic microbiological and standard biochemical methods. Stock cultures were kept frozen at −75 °C in a brain heart infusion broth containing 25 % glycerol. Two *S. epidermidis* reference strains were used as controls: the well-known slime-producing strain ATCC 35984 (RP62A) and the non-slime-producing strain ATCC 12228.

### Biofilm model

Biofilms were studied using the static microtitre plate model established by Christensen et al. [5]. Ketorolac tris salt (Sigma) was prepared as 2-M stock solutions in PBS.

The *S. epidermidis* isolates were prepared as follows: an overnight culture grown in trypticase soy broth (TSB) at 37 °C was diluted to 1:100 in TSB. A total of 200 μl of these cell suspensions was transferred to a sterile 96-well polystyrene U-bottom microtitre plate in TSB alone (control group) or in fresh TSB with 13 mM ketorolac added (ketorolac group). The plates were incubated aerobically for 24 h at 37 °C. Media and planktonic cells were removed. Adherent biofilm was fixed with 95 % ethanol and was stained with 100 μl of 1 % (*w*/*v*) crystal violet for 5 min. Then, unbound crystal violet was removed, and the wells were washed three times with 300 μl of sterile distilled water. The water was then cleared and the microtitre plate was air-dried for 2 h. The mean optical density (OD) was used for quantification using a routine microtitre plate reader at a wavelength of 570 nm. All biofilm experiments were performed three times for each isolate to minimize the variability in OD measurements. To measure changes in the thickness of the biofilm, a ratio was calculated of the biofilm OD of the isolate incubated with ketorolac against the biofilm OD of the same isolate without ketorolac: (OD_R_ = OD of the treated biofilm/OD of untreated biofilm). The significance of differences (*P* < 0.05) was assessed using the Mann–Whitney *U* test.

## Results

**Fig. 1:**
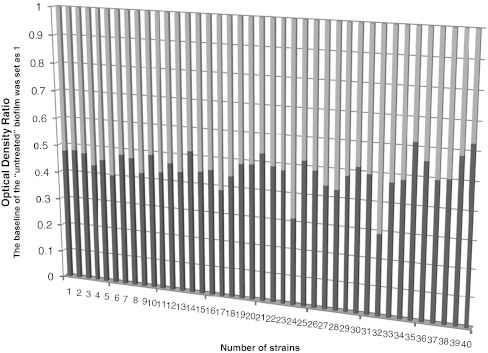
The graph shows that ketorolac reduced the biofilm of all strains tested expressed as OD ratio

Biofilm ODs of the *S. epidermidis* isolates untreated and treated with ketorolac were significantly different (0.335 ± 0.06 versus 0.158 ± 0.03, respectively; mean ± SD; *P* < 0.001). Ketorolac reduced the *S. epidermidis* biofilm by 47.6 % or 0.476 ± 0.07 (mean ± SD) expressed as OD ratio (Table 1). This effect was present in the 40 clinical isolates of *S. epidermidis* tested (Fig. 1).

**Table 1:** Effect of ketorolac on *S. epidermidis* biofilm formation by microplate assay

Strain	OD_570_ control (mean ± SD)	OD_570_ (ketorolac) (mean ± SD)	OD_R_
1	0.382 ± 0.06	0.183 ± 0.03	0.479
2	0.238 ± 0.01	0.115 ± 0.01	0.483
3	0.305 ± 0.01	0.145 ± 0.06	0.475
4	0.297 ± 0.12	0.129 ± 0.03	0.434
5	0.365 ± 0.04	0.167 ± 0.07	0.457
6	0.284 ± 0.02	0.115 ± 0.04	0.405
7	0.267 ± 0.08	0.129 ± 0.01	0.483
8	0.329 ± 0.05	0.156 ± 0.08	0.474
9	0.396 ± 0.05	0.168 ± 0.05	0.424
10	0.412 ± 0.06	0.203 ± 0.09	0.493
11	0.265 ± 0.06	0.115 ± 0.04	0.434
12	0.423 ± 0.09	0.198 ± 0.11	0.469
13	0.398 ± 0.04	0.176 ± 0.06	0.442
14	0.256 ± 0.02	0.133 ± 0.04	0.519
15	0.411 ± 0.11	0.185 ± 0.02	0.450
16	0.357 ± 0.07	0.164 ± 0.04	0.459
17	0.276 ± 0.03	0.108 ± 0.05	0.391
18	0.351 ± 0.08	0.139 ± 0.01	0.444
19	0.397 ± 0.06	0.195 ± 0.01	0.491
20	0.408 ± 0.03	0.201 ± 0.03	0.493
21	0.325 ± 0.09	0.174 ± 0.04	0.535
22	0.306 ± 0.05	0.151 ± 0.04	0.493
23	0.409 ± 0.08	0.197 ± 0.08	0.482
24	0.435 ± 0.12	0.138 ± 0.01	0.317
25	0.298 ± 0.07	0.156 ± 0.05	0.523
26	0.254 ± 0.04	0.125 ± 0.02	0.492
27	0.306 ± 0.02	0.136 ± 0.04	0.444
28	0.335 ± 0.09	0.145 ± 0.02	0.433
29	0.408 ± 0.12	0.198 ± 0.13	0.485
30	0.312 ± 0.08	0.162 ± 0.04	0.519
31	0.319 ± 0.08	0.159 ± 0.09	0.498
32	0.397 ± 0.09	0.117 ± 0.01	0.295
33	0.243 ± 0.03	0.116 ± 0.03	0.477
34	0.268 ± 0.12	0.131 ± 0.05	0.489
35	0.305 ± 0.05	0.189 ± 0.06	0.620
36	0.315 ± 0.02	0.176 ± 0.02	0.559
37	0.403 ± 0.1	0.201 ± 0.05	0.5
38	0.409 ± 0.04	0.207 ± 0.06	0.506
39	0.298 ± 0.05	0.175 ± 0.06	0.587
40	0.257 ± 0.04	0.162 ± 0.07	0.630
All strains	0.335 ± 0.06	0.158 ± 0.03	0.476 ± 0.07

## Discussion

The incidence of post-cataract surgery endophthalmitis varies considerably in the medical literature. An incidence of 1.11 per 1,000 surgeries in the USA has been reported [6], 0.48 per 1,000 surgeries in Sweeden [7], 1.4 per 1,000 surgeries in Canada [8], and 0.49 per 1,000 surgeries in Brazil [9]. Visual outcome after endophthalmitis is generally poor; half the patients of the Endophthalmitis Vitrectomy Study achieved a visual acuity of 20/40 or better by 9 months after treatment [10].

Microorganisms from the ocular and periocular surface may enter the anterior chamber during phacoemulsification, with reported rates of anterior chamber contamination as high as 21 % [11]. In the Endophthalmitis Vitrectomy Study, 70 % of microbiological isolates contained coagulase-negative micrococci [12]. The isolates involved are usually normal flora of the surface of the eye and surrounding mucosa, such as *S. epidermidis*, which are generally not highly virulent. *S. epidermidis* has been isolated from 70 % of normal eyes [13].

Biofilm is a microbially derived sessile community characterized by cells that are irreversibly attached to a substratum or interface or to each other, and are embedded in a matrix of extracellular polymeric substances that they have produced exhibiting an altered phenotype with respect to growth rate and gene transcription [14]. Formation of *S. epidermidis* biofilm is one of the most important virulence factors. Strains isolated from septic patients and prosthetic infections have the *ica* genes which mediate biofilm formation, and the *mec*A gene which is related to methicillin resistance. However, saprophytic isolates usually do not have these markers of virulence [15, 16].

This in vitro study shows that ketorolac reduces the formation of biofilm by 47.6 % on all the strains tested. The mechanism by which ketorolac decreased biofilm production was not addressed in this study, and further research is needed for elucidation. The concentration of ketorolac used in our experiment is equivalent to approximately 0.5 % ketorolac, the formulation present in commercially available products. Nonsteroidal anti-inflammatory drugs (NSAIDs) like salicylic acid and ketorolac can prevent adhesion and bacterial colonization of contact lenses [3]. Salicylic acid inhibited the production of teichoic acid, slime-associated proteins, and polysaccharide/adhesin production by *S. epidermidis* [17]. The adherence of bacteria to intraocular lenses (IOLs) during implantation and colonization of IOLs appears to have a role in the pathogenesis of postoperative endophthalmitis [18, 19]. Herein is the importance for reducing biofilm production.

Most ophthalmic medications contain benzalkonium chloride as preservative. It has been shown that the presence of benzalkonium chloride at the minimal inhibitory is able to inhibit biofilm formation. However, it was able to induce biofilm development for the *S. epidermidis* at sub-MIC [20].

The failure of antibiotic treatment of biofilm-associated infections has led to a search for additive measures to eradicate bacteria within biofilms. Our findings support the hypothesis that the use of ketorolac in the perioperative period of cataract surgery may decrease the virulence of *S. epidermidis*, the most common microorganism involved in post-cataract endophthalmitis. Further studies are needed to prove this effect in vivo.

## Conclusion

Ketorolac significantly reduces the formation of biofilm by strains of *S. epidermidis* that previously caused endophthalmitis. This effect may play a role in a new prophylactic strategy for postoperative endophthalmitis.
